# Temporal Trends in Suicidal Ideation and Attempts Among US Adolescents by Sex and Race/Ethnicity, 1991-2019

**DOI:** 10.1001/jamanetworkopen.2021.13513

**Published:** 2021-06-14

**Authors:** Yunyu Xiao, Julie Cerel, J. John Mann

**Affiliations:** 1 School of Social Work, Indiana University–Purdue University, Indianapolis; 2 School of Social Work, Indiana University, Bloomington; 3 College Social Work, University of Kentucky, Lexington; 4 Department of Psychiatry and Radiology, Columbia University Irving Medical Center, Columbia University, New York, New York; 5 Division of Molecular Imaging and Neuropathology, New York State Psychiatric Institute, New York

## Abstract

**Importance:**

Disparities by sex and racial/ethnic group in suicide death rates are present in US adolescents. Whether disparities in suicide death extend to groups targeted for suicide prevention efforts, namely, those with suicidal ideation or nonfatal suicide attempts, is unknown.

**Objective:**

To examine differences in temporal trends between suicidal ideation and suicide attempts in US adolescents from 1991 through 2019 by sex and race/ethnicity subgroups.

**Design, Setting, and Participants:**

A cross-sectional analysis of the national Youth Risk Behavior Survey, weighted to represent US adolescents from 1991 to 2019, included 183 563 US high-school students in grades 9 to 12. Data were analyzed from September 16, 2020, through April 12, 2021.

**Exposures:**

Calendar year, sex, race/ethnicity, and interactions of sex and race/ethnicity.

**Main Outcomes and Measures:**

Survey-weighted prevalence estimates, annual percentage changes (APCs) and average APC in the survey-weighted prevalence of suicidal ideation and nonfatal suicide attempts, constructed from self-reported suicidal ideation, plan, and attempts in each survey year, by sex, race/ethnicity (non-Hispanic White, non-Hispanic Black, Hispanic, non-Hispanic Asian or Pacific Islander and Native Hawaiian, American Indian/Alaska Native), and their interactions (sex × race/ethnicity).

**Results:**

In 183 563 (unweighted) included adolescents (mean [SD] age, 16.07 [1.23] years; 94 282 females [weighted percentage, 49.4%; 95% CI, 48.8%-50.1%]), the prevalence of suicidal ideation decreased from 1991 to 2019 (from 19.4% to 15.8%; 95% CI, 0.7%-0.9%), whereas the prevalence of nonfatal suicide attempts increased from 1991 to 2019 (from 7.3% to 8.9%; 95% CI, 1.0%-1.4%). Joinpoint regression indicated a −3.1% (95% CI, −3.7% to −2.6%) annual decrease in suicidal ideation between 1991 and 2009, followed by a 3.4% annual increase (95% CI, 1.9% -4.8%) between 2009 and 2019. Decreasing followed by increasing trends in suicidal ideation showed modestly different turning points in female (1991-2009, 2009-2019), White (1991-2009, 2009-2019), Hispanic (1991-2007, 2007-2019), and Black (1991-2005, 2005-2019) adolescents. Although no significant trends were observed in suicide attempts from 1991 through 2019, male (68.4% increase; 95% CI, 0.2% -1.2%) and Black (79.7% increase; 95% CI, 0.1%-1.5%) adolescents had greater increases in the prevalence of suicide attempts. Interaction of sex and race/ethnicity revealed increases in suicidal ideation in White females from 2009 to 2019 (APC, 4.3%; 95% CI, 1.5%-7.1%), Black females from 2005 to 2019 (APC, 3.4%; 95% CI, 1.4%-5.4%), and Hispanic females from 2009 to 2019 (APC, 3.3%; 95% CI, 1.0%-5.6%) and suicide attempts in White females from 2009 to 2019 (APC, 3.1%; 95% CI, 0.3%-6.0%).

**Conclusions and Relevance:**

The findings of this study show apparent sex and racial/ethnic differences in trends in suicidal ideation and suicide attempts. Increases in suicidal ideation since 2009 were observed in female individuals; changes in male and Black adolescents represented the largest increase in the prevalence of suicide attempts between 1991 and 2019. Evidence-based suicide prevention programs need to be tailored by sex and race/ethnicity, calling for greater diversification of health care system, school, and community prevention approaches.

## Key Points

**Question:**

Are temporal trends in suicidal ideation and attempts among US adolescents different across sex and race/ethnicity from 1991 through 2019?

**Findings:**

In this cross-sectional study of 183 563 adolescents in the US, a decreasing temporal trend in suicidal ideation changed to an increase, with different turning points for female (2009), White (2009), Hispanic (2007), and Black (2005) adolescents. Male and Black youths had nonsignificant changes in suicidal ideation, but the greatest increase in the prevalence of nonfatal suicide attempts.

**Meaning:**

The results of this study suggest that sex and racial/ethnic disparities in temporal trends in adolescents with suicidal ideation and attempts call for greater diversification in suicide prevention.

## Teaser

This cross-sectional study analyses temporal trends to assess changes in suicidal ideation and nonfatal suicide attempts among US adolescents from 1991 through 2019.

## Introduction

Suicide is the second leading cause of death in youths aged 12 to 18 years in the US.^1^ In 2019, 2090 (7.1 per 100 000) adolescents in this age group died by suicide.^2^ Visits to hospital emergency departments due to suicidal ideation or attempts in youths aged 15 to 19 years are increasing,^3^ with the largest increase observed in Black, Hispanic, and female adolescents between 2008 and 2015.^4,5^ Monitoring the temporal trends of suicidal behaviors is important to guide development of effective suicide prevention.^6,7,8,9^ In particular, detecting changes in temporal trends in subgroups and when the risk of suicide is greater in those groups can identify high-risk populations for targeted intervention and guide hypothesis generation and testing.^10^

Previous studies have reported increasing rates of suicide death,^11,12^ nonfatal suicidal ideation, plan attempts, and injury due to attempts.^12,13,14,15,16^ However, most work to date did not separate youths who attempted suicide from those who had suicidal ideation but no attempts.^17^ This step is needed, because theoretical and empirical evidence suggests that the risk factors associated with suicidal ideation and suicide attempts are likely to differ.^18,19^ From a public health perspective, knowing whether and how the trends differ between suicidal ideation and attempts is an important component of surveillance.^7^ Increasing trends in specific suicidal behaviors across demographic groups may signal emerging risk factors or high-risk groups that warrant targeted suicide prevention.^20^

There are increasing data on sex, racial/ethnic, and intersectionality disparities in suicidal behaviors.^12,13,21,22,23^ Black adolescents have been reported to be the only racial/ethnic group to show increasing suicide attempts between 1991 and 2017.^17^ Another study found a narrowing sex gap in youth suicide death between 1975 and 2016.^12^ Although informative, the existing literature lacks information about any turning points of the trends in suicidal ideation and nonfatal suicide attempts and possible sex and racial/ethnic disparities. Identifying turning points can help in understanding the possible influence of structural inequalities and socioeconomic and political changes on sex- and race/ethnicity-specific suicide trends^24^ (eFigure 1 in the Supplement).

To our knowledge, the present study provides the first nationally representative analysis of temporal trends to examine changes in suicidal ideation and nonfatal suicide attempts among US adolescents from 1991 through 2019. A secondary objective was to identify the demographic subgroups of adolescents whose trends in terms of suicidal ideation and nonfatal suicide attempts indicate a need to be prioritized for interventions.

## Methods

### Data and Participants

Since 1991, the national Youth Risk Behavior Survey (YRBS) has been conducted biannually. Each cross-sectional survey cycle uses a 3-stage cluster sampling design to produce a nationally representative sample of high school students in grades 9 to 12 among public and private schools in the 50 states and the District of Columbia. Written informed consent was obtained from the parent or legal guardian of adolescents. Students completed the self-administered, computer-scannable questionnaire anonymously and voluntarily. No compensation was provided. The Centers for Disease Control and Prevention Institutional Review Board approved each independent protocol of the national YRBS.^25^ This study followed the Strengthening the Reporting of Observational Studies in Epidemiology (STROBE) reporting guideline for cross-sectional studies.

This study pooled data from the 1991-2019 YRBS, across which unweighted sample sizes ranged from 10 904 to 16 410. Data were analyzed from September 16, 2020, through April 12, 2021. School response rates ranged from 75% to 81%; student response rates ranged from 81% to 90%, and overall response rates for the surveys ranged from 60% to 71%. More details regarding sampling strategies and reliabilities of the YRBS survey items are reported elsewhere.^26^ The analytic sample included 183 563 participants who answered the questions reporting past-year suicide ideation, plan, and/or attempt. Adolescents who had missing information in all 3 suicidal behavior questions were excluded.

### Measures

The YRBS assessed 3 self-reported questions related to suicidal behaviors: (1) suicide ideation (During the past 12 months, did you ever seriously consider attempting suicide?), (2) suicide plan (During the past 12 months, did you make a plan about how you would attempt suicide?), and (3) suicide attempts (During the past 12 months, how many times did you actually attempt suicide?). Response options were dichotomized into yes or no. These questions demonstrated substantial reliability in earlier studies.^27,28,29^

All respondents who reported suicidal ideation or suicide plan but denied suicide attempts were coded as suicidal ideation (n = 24 435). Respondents who reported 1 or more suicide attempts, regardless of their answers to ideation or plan, were coded as suicide attempts (n = 16 497). We focused on nonfatal suicidal behaviors in this study. The suicidal ideation and suicide attempts groups are not mutually exclusive.

The YRBS asks 2 self-report questions about race and Hispanic heritage. Consistent with previous research,^30^ the 6 categories for race/ethnicity were non-Hispanic White; non-Hispanic Black; non-Hispanic Asian or Pacific Islander and Native Hawaiian (API); Hispanic; American Indian/Alaska Native (AI/AN), and non-Hispanic multiple races. Sex groups were self-reported biological sex. Student grade levels were included as controlled variables. Intersectional terms of sex and race/ethnicity were constructed. Prevalence of suicidal ideation and suicide attempts were calculated for the total sample and by sex, race/ethnicity, and sex × race/ethnicity subgroups, adjusting for survey weights and complex sample design.

### Statistical Analysis

Weighted prevalence of suicidal ideation and suicide attempts among the total sample and separately by sex and race/ethnicity from 1991 through 2019 was calculated using Stata, version 16 (StataCorp LLC). To present the magnitude of the changes, the percent differences in the prevalence of suicidal ideation and suicide attempts were calculated using the formula [(rate in 2019 − rate in 1991)/rate in 1991] × 100. Changes in prevalence show the magnitude of changes in the population. Joinpoint, version 4.9.0.0 (National Cancer Institute), was used to estimate piecewise log-linear trends in the survey-weighted prevalence estimates of suicidal ideation and suicide attempts over time. After fitting the regression to the natural logarithm of prevalence using calendar year as a regressor, turning points of change in trends (joinpoints) were identified by a series of Monte Carlo permutation-based tests and model selection.^31^ Annual percentage change (APC) with 95% CI for each identified trend was estimated. Average APC, a single summary measure, was used to describe the average rate of change over the entire 1991 to 2019 study period. The APC and average APC help identify the time at which the trend in suicidal ideation and attempts changed significantly compared with the trend in previous years. Significant slopes (95% CIs not crossing 0; *P* < .05) indicated increasing and decreasing trends, whereas nonsignificant APC slopes (95% CIs crossing 0; *P* > .05) indicated that the prevalence was stable.

Trends were stratified by sex, race/ethnicity, and intersectional terms. Disparities in slopes of suicidal subgroups across sex, race/ethnicity, and intersectionality were tested using parallel pairwise comparisons that detected differences in APCs between 2 models.^32^ All analyses adjusted for the complex sampling design and survey weight to obtain the US nationally representative estimates, accounting for nonresponse and oversampling of Black and Hispanic students. Trends are presented as a straight line fitted over the full period based on a simple log-linear model if no statistically significant change in trends emerged. If a significant change in the slope of the trend was noted, linear segments connected between 2 years (ie, joinpoints) are presented. Statistical significance was determined by a 2-sided *P* value <.05 with Wald χ^2^ tests using design-adjusted coefficient variance-covariance matrices.

## Results

### Overall Trends and Turning Points

**Figure 1.**
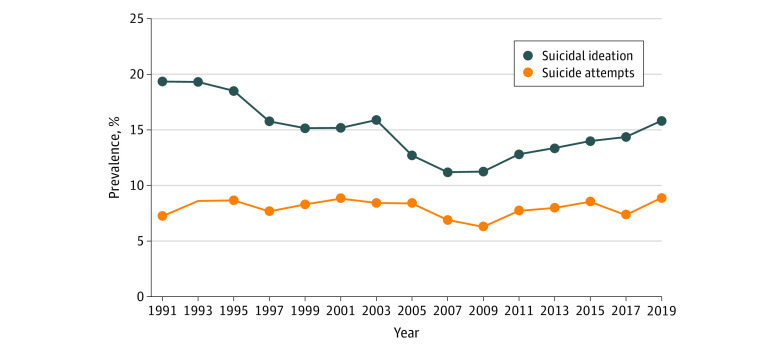
Prevalence of Suicidal Ideation and Suicide Attempts Among US Adolescents, 1991-2019

Of 183 563 adolescents (mean [SD] age, 16.07 [1.23] years), 94 282 (unweighted; weighted percentage, 49.4% [95% CI, 48.8%-50.1%]) were female, 89 281 (unweighted; weighted percentage, 50.5% [95% CI, 49.9%-51.2%]) were male, 81 838 (unweighted; weighted percentage, 64.0% [95% CI, 62.3%-65.6%]) were non-Hispanic White adolescents, 37 009 (unweighted; weighted percentage, 12.5% [95% CI, 11.6%-13.4%]) were non-Hispanic Black adolescents, 49 554 (unweighted; weighted percentage, 15.5% [95% CI, 14.4%-16.6%]) were Hispanic adolescents, 7875 (unweighted; weighted percentage, 4.0% [95% CI, 3.5%-4.5%]) were non-Hispanic API adolescents, 1997 (unweighted; weighted percentage, 0.7% [95% CI, 0.6%-0.9%]) were non-Hispanic AI/AN adolescents, and 5290 (unweighted; weighted percentage, 3.3% [95% CI, 3.0%-3.7%]) were non-Hispanic multiple races or other race. In the total sample (Figure 1), the prevalence of suicidal ideation without attempts decreased by 18.2% from 1991 to 2019 (from 19.4% to 15.8%; 95% CI, 0.7%-0.9%). The prevalence of nonfatal suicide attempts increased by 22.5% from 1991 to 2009 (from 7.3% to 8.9%; 95% CI, 1.0%-1.4%).

**Figure 2.**
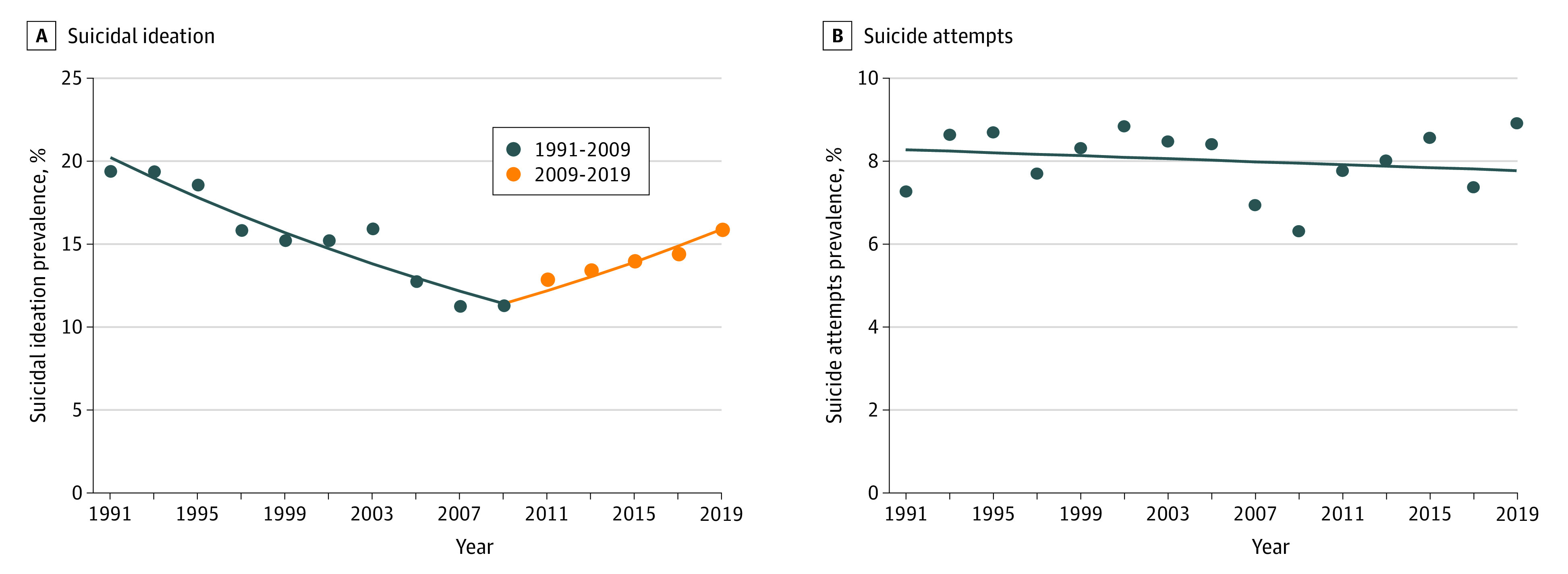
Trends in Suicidal Ideation and Suicide Attempts Among the Total Sample, 1991-2019

Joinpoint regression identified a decreasing trend in suicidal ideation from 1991 to 2019 among the total sample (average APC, −0.9%; 95% CI, −1.4% to −0.3%), with 2009 the turning point of a decreasing-increasing trend observed in suicidal ideation (Table, Figure 2). In the total sample, the prevalence of suicidal ideation decreased from 1991 to 2009 (APC, −3.1%; 95% CI, −3.7% to −2.6%), followed by an increase from 2009 to 2019 (APC, 3.4%; 95% CI, 1.9%-4.8%). Trends in nonfatal suicide attempts in the total sample were not significant and no turning points were observed (APC, −0.2%; 95% CI, −0.9% to 0.5%) (Table, Figure 2).

**Table.** Annual Percentage Changes in Suicidal Ideation and Suicide Attempts Among US Adolescents, 1991-2019^a^

Demographic characteristic	Average APC, % (95% CI)	Segment 1	APC, % (95% CI)	Segment 2	APC, % (95% CI)	Segment 3	APC, % (95% CI)
**Total**							
Suicidal ideation	−0.9 (−1.4 to −0.3)	1991-2009	−3.1 (−3.7 to −2.6)	2009-2019	3.4 (1.9 to 4.8)	NA	NA
Suicide attempts	−0.2 (−0.9 to 0.5)	1991-2019	−0.2 (−0.9 to 0.5)	NA	NA	NA	NA
**Sex**							
Suicidal ideation							
Female	−0.7 (−1.3 to −0.1)	1991-2009	−3.2 (−3.8 to −2.7)	2009-2019	4.0 (2.5 to 5.6)	NA	NA
Male	−1.3 (−2.6 to 0.0)	1991-2007	−3.3 (−4.9 to −1.6)	2007-2019	1.4 (−1.2 to 4.0)	NA	NA
Suicide attempts							
Female	−0.6 (−1.3 to 0.1)	1991-2019	−0.6 (−1.3 to 0.1)	NA	NA	NA	NA
Male	0.6 (−0.2 to 1.5)	1991-2019	0.6 (−0.2 to 1.5)	NA	NA	NA	NA
**Race/ethnicity**							
Suicidal ideation							
White	−1.0 (−1.8 to −0.2)	1991-2009	−3.4 (−4.2 to −2.7)	2009-2019	3.6 (1.3 to 5.9)	NA	NA
Black	−0.1 (−1.4 to 1.2)	1991-2005	−3.3 (−5.2 to −1.3)	2005-2019	3.1 (1.0 to 5.4)	NA	NA
Hispanic	−0.7 (−1.7 to 0.3)	1991-2007	−2.6 (−4.1 to −1.2)	2007-2019	1.9 (0.2 to 3.6)	NA	NA
API	−1.1 (−1.9 to −0.2)	1991-2019	−1.1 (−1.9 to −0.2)	NA	NA	NA	NA
AI/AN	1.2 (−0.5 to 2.9)	1991-2019	1.2 (−0.5 to 2.9)	NA	NA	NA	NA
Suicide attempts							
White	−0.5 (−1.4 to 0.4)	1991-2019	−0.5 (−1.4 to 0.4)	NA	NA	NA	NA
Black	0.6 (−0.1 to 1.4)	1991-2019	0.6 (−0.1 to 1.4)	NA	NA	NA	NA
Hispanic	−1.1 (−2.1 to −0.1)	1991-2019	−1.1 (−2.1 to −0.1)	NA	NA	NA	NA
API	−1.1 (−2.7 to 0.5)	1991-2019	−1.1 (−2.7 to 0.5)	NA	NA	NA	NA
AI/AN	−1.4 (−3.4 to 0.7)	1991-2019	−1.4 (−3.4 to 0.7)	NA	NA	NA	NA
**Sex × race/ethnicity**							
Suicidal ideation							
Female							
White	−0.9 (−1.9 to 0.1)	1991-2009	−3.6 (−4.5 to −2.8)	2009-2019	4.3 (1.5 to 7.1)	NA	NA
Black	0.1 (−1.1 to 1.3)	1991-2005	−3.1 (−4.9 to −1.2)	2005-2019	3.4 (1.4 to 5.4)	NA	NA
Hispanic	−0.2 (−1.1 to 0.7)	1991-2009	−2.1 (−3.1 to −1.1)	2009-2019	3.3 (1.0 to 5.6)	NA	NA
API	−1.5 (−2.7 to −0.2)	1991-2019	−1.5 (−2.7 to −0.2)	NA	NA	NA	NA
AI/AN	2.4 (−0.6 to 5.6)	1991-2019	2.4 (−0.6 to 5.6)	NA	NA	NA	NA
Male							
White	−1.5 (−2.5 to −0.4)	1991-2007	−3.5 (−4.7 to −2.2)	2007-2019	1.3 (−0.9 to 3.5)	NA	NA
Black	−0.5 (−2.2 to 1.3)	1991-2019	−0.5 (−2.2 to 1.3)	NA	NA	NA	NA
Hispanic	−1.0 (−1.9 to 0.0)	1991-2019	−0.5 (−2.2 to 1.3)	NA	NA	NA	NA
API	−0.2 (−1.6 to 1.1)	1991-2019	−1.0 (−1.9 to 0.0)	NA	NA	NA	NA
AI/AN	0.1 (−2.2 to 2.5)	1991-2019	−0.2 (−1.6 to 1.1)	NA	NA	NA	NA
Suicide attempts							
Female							
White	−0.4 (−2.8 to 1.9)	1991-2003	−0.5 (−2.5 to 1.5)	2003-2009	−6 (−16.4 to 5.8)	2009-2019	3.1 (0.3 to 6.0)
Black	0.5 (−0.4 to 1.4)	1991-2019	0.5 (−0.4 to 1.4)	NA	NA	NA	NA
Hispanic	−1.5 (−2.5 to −0.5)	1991-2019	−1.5 (−2.5 to −0.5)	NA	NA	NA	NA
API	−1.4 (−3.2 to 0.5)	1991-2019	−1.4 (−3.2 to 0.5)	NA	NA	NA	NA
AI/AN	−0.2 (−2.9 to 2.6)	1991-2019	−0.2 (−2.9 to 2.6)	NA	NA	NA	NA
Male							
White	0.9 (−0.3 to 2.2)	1991-2019	0.9 (−0.3 to 2.2)	NA	NA	NA	NA
Black	0.7 (−0.5 to 2.0)	1991-2019	0.7 (−0.5 to 2.0)	NA	NA	NA	NA
Hispanic	1.6 (−0.9 to 4.2)	1991-1995	0.7 (−0.5 to 2.0)	1995-2019	−0.8 (−2.1 to 0.5)	NA	NA
API	−1.2 (−3.3 to 1.0)	1991-2019	17.7 (−2.0 to 41.4)	NA	NA	NA	NA
AI/AN	−0.6 (−4.4 to 3.5)	1991-2019	−1.2 (−3.3 to 1.0)	NA	NA	NA	NA

Abbreviations: AI/AN, non-Hispanic American Indian/Alaska Native; APC, annual percentage change; API, non-Hispanic Asian or Pacific Islander and Native Hawaiian; NA, not applicable.

^a^ Segments were chosen by joinpoint regression.

### Differences by Sex

**Figure 3.**
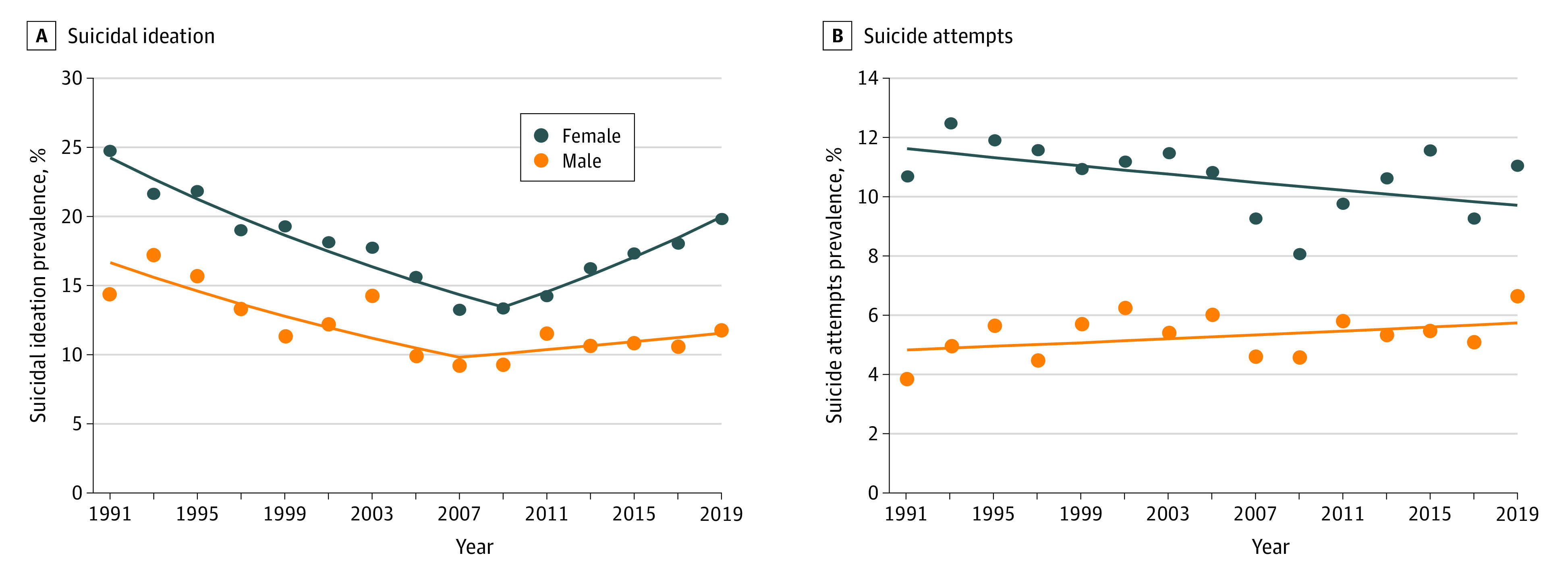
Trends in Suicidal Ideation and Suicide Attempts by Sex, 1991-2019

The prevalence of suicidal ideation in females and males decreased comparably between 1991 and 2019 (18.5% for females, 21.8% for males) (eFigure 2 in the Supplement). Joinpoint regression (Table) estimated that suicidal ideation in females decreased from 1991 to 2009 (APC, −3.2%; 95% CI, −3.8% to −2.7%) and increased from 2009 to 2019 (APC, 4.0%; 95% CI, 2.5%-5.6%). Suicidal ideation in males decreased from 1991 to 2007 (APC, −3.3%; 95% CI, −4.9% to −1.6%) and remained stable thereafter (APC, 1.4%; 95% CI, −1.2% to 4.0%) (Figure 3). APCs of suicidal ideation in males and females were parallel, but there were statistically significant differences in APCs in nonfatal suicide attempts by sex (eFigure 2 in the Supplement). Between 1991 and 2019, the APC in female adolescents was −0.6% (95% CI, −1.3% to 0.1%) and, in male adolescents, 0.6% (95% CI, −0.2% to 1.5%). The increasing prevalence of nonfatal suicide attempts was largely attributable to male adolescents (68.4%; 95% CI, 0.2%-1.2%), with the rate 20 times greater compared with females (3.4%; 95% CI, −0.1% to 0.2%) between 1991 and 2019 (eFigure 2 in the Supplement).

### Differences by Race/Ethnicity

**Figure 4.**
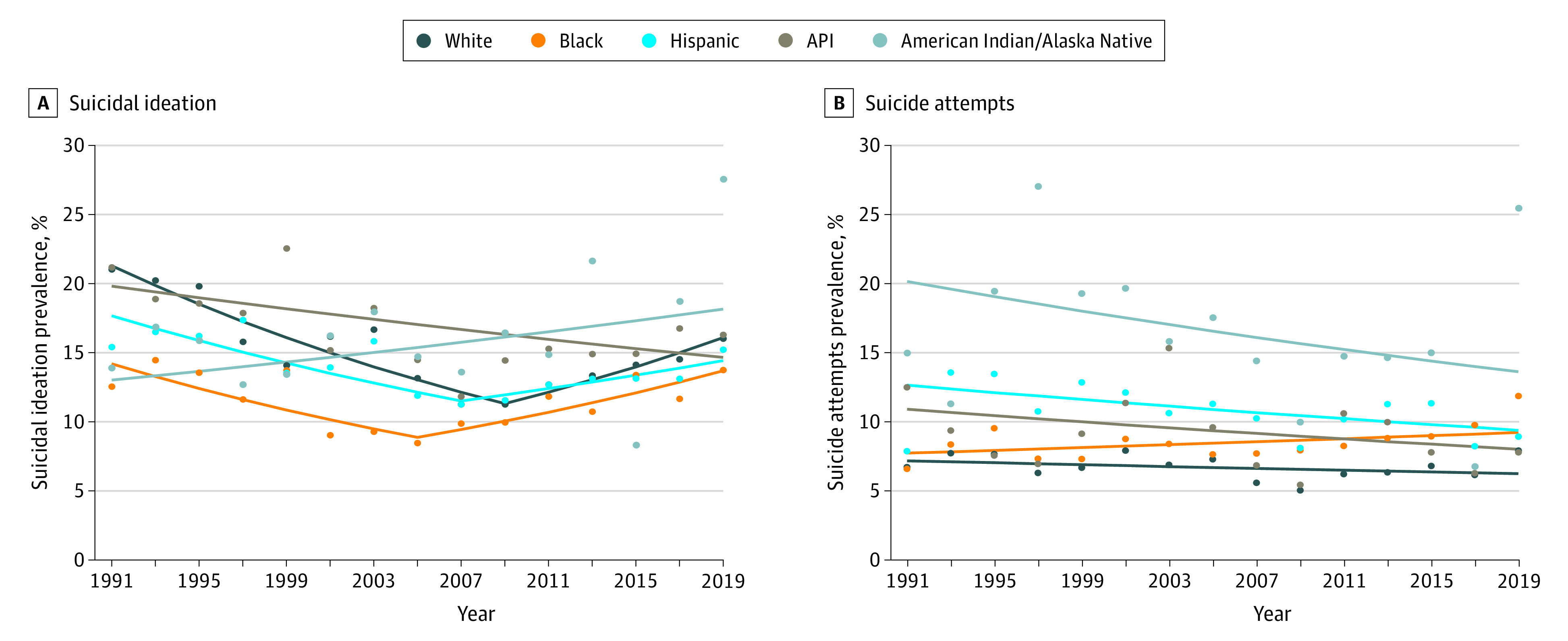
Trends in Suicidal Ideation and Suicide Attempts by Race/Ethnicity, 1991-2019 API indicates non-Hispanic Asian or Pacific Islander and Native Hawaiian.

Suicidal ideation in Black (9.5%) and AI/AN (98.1%) adolescents has increased from 1991 through 2019 compared with a decreasing prevalence in other races/ethnicities (White, −23.8%; Hispanic, −1.2%; API, −23.1%) (eFigure 3 in the Supplement). Suicidal ideation in White, Black, and Hispanic adolescents showed decreasing and increasing trends over the study period but with different turning points: 2009 for White adolescents (from APC, −3.4%; 95% CI, −4.2% to −2.7% to APC, 3.6%; 95% CI, 1.3%-5.9%), 2005 for Black adolescents (from APC, −3.3%; 95% CI, −5.2% to −1.3% to APC, 3.1%; 95% CI, 1.0%-5.4%), and 2007 for Hispanic adolescents (from APC, −2.6%; 95% CI, −4.1% to −1.2% to APC, 1.9%; 95% CI, 0.2%-3.6%). Suicidal ideation in API adolescents decreased from 1991 to 2019 (APC, −1.1%; 95% CI, −1.9% to −0.2%). There were statistically significant differences in APCs in suicidal ideation across racial/ethnic groups (eFigure 2 in the Supplement; Table, Figure 4).

Black adolescents had the highest increase in the prevalence of suicide attempts between 1991 and 2019 (79.7%; 95% CI, 0.1%-1.5%) compared with all other races/ethnicities (White, 17.8%; Hispanic, 13.4%; API, −37.6%; AI/AN, 70.0%). Joinpoint regression estimated a downward trend in suicide attempts in Hispanic adolescents from 1991 to 2019 (APC, −1.1%; 95% CI, −2.1% to −0.1%). No changes were detected in other racial/ethnic groups, despite the only positive APC (0.6%; 95% CI, −0.1% to 1.4%) observed in Black adolescents (White: APC, −0.5%; 95% CI, −1.4% to 0.4%; API: APC, −1.1%; 95% CI, −2.7% to 0.5%; AI/IN: APC, −1.4%; 95% CI, −3.4% to 0.7%) (Table, Figure 4). Parallel comparisons in trends in suicide attempts indicated different APCs between White and Black adolescents and Black and Hispanic adolescents.

### Differences by Sex and Race/Ethnicity

Stratifying by both sex and race/ethnicity revealed further subgroup differences. During 1991-2019, Black (13.4%) and AI/AN (32.6%) females showed an increasing prevalence of suicidal ideation and other female subgroups had decreasing rates. In addition, AI/AN (204.0%) and Black (20.8%) males had higher increasing rates in nonfatal suicide attempts compared with other male racial/ethnic groups (eFigure 4 in the Supplement). Suicidal ideation in White females decreased during 1991-2009 (APC, −3.6%; 95% CI, −4.5% to −2.8%) and increased during 2009-2019 (APC, 4.3%; 95% CI, 1.5%-7.1%). Similar decreasing and increasing trends were observed in suicidal ideation in Black and Hispanic females with different turning points. Suicidal ideation in API females (APC, −1.5%; 95% CI, −2.7% to −0.2%) decreased over the entire study period (Table; eFigure 5 in the Supplement).

Black males had the highest increase in suicide attempts (162.4%) compared with all other sex and racial/ethnic subgroups (White females, −9.3%; Black females, 62.1%; Hispanic females, 3.0%; API females, −43.2%; AI/AN females, 65.2%; White males, 92.7%; Hispanic males, 50.3%; API males, 30.0%; AI/AN males, 86.7%) from 1991 to 2019. In the past decade (2009 to 2019), suicide attempts increased by 3.1% annually (95% CI, 0.3%-6.0%) among White females, whereas there was a consistent decrease in suicide attempts among Hispanic females from 1991 to 2019 (APC, −1.5%; 95% CI, −2.5% to −0.5%). The prevalence of suicide attempts remained stable among other sex and racial/ethnic subgroups.

## Discussion

To our knowledge, this is the first study to examine and compare the temporal trends in suicidal ideation and nonfatal suicide attempts across sex and race/ethnicity during 1991-2019 using a nationally representative sample of US adolescents. The core finding is the sex and race/ethnic disparities in the 22.5% increase in the prevalence of US adolescent suicide attempts from 1991 to 2019, and the 18.2% decrease in prevalence of suicidal ideation over the same period. We detected the largest percentage increase in suicide attempts in male and Black adolescents. Trends in suicidal ideation among females, particularly among non-Hispanic Black, non-Hispanic White, and Hispanic females, showed a V-shape (decreasing-increasing) change across the entire study period (1991-2019), with different turning points (2005 in non-Hispanic Black, 2007 in non-Hispanic White, 2009 in Hispanic females). Trends in suicide attempts were stable, except for a significant increasing trend among non-Hispanic White female adolescents from 2009 to 2019. Our findings highlight emerging sex and racial/ethnic disparity in the epidemiologic factors associated with US adolescent suicide.

Our finding of the substantial increase in suicide attempts parallels the increase in emergency department visits for nonfatal self-inflicted injuries among youths and young adults.^4,5,33,34^ We detected a larger increase in male adolescents attempting suicide than in their female counterparts from 1991 through 2019, which is consistent with previous studies on suicide death rates among male children and youths aged 5 to 18 years between 1991 and 2017.^14,23,35^ Possible male-specific risk factors include greater access to firearms at home,^36^ less predisposition to seek help,^37^ and a greater tendency to adopt avoidance strategies to cope with emotional problems.^38^ Other studies, however, have revealed greater increases in emergency department visits for suicide attempts among females than males between 2001-2015,^4^ 2008-2015,^33^ and 2011-2015.^5^ Owing to the self-reported nature of the suicide attempt questions in our data, we cannot rule out the possibility of male youths becoming more willing to self-disclose in more recent cohorts. More research is warranted to examine emerging risk factors for these groups.

Of public health concern, suicide attempts among Black adolescents have increased rapidly, accelerating by 79.7% from 1991 to 2019. This finding is consistent with prior research on increasing self-reported suicidal behaviors between 1991 and 2018,^17^ emergency department visits for suicide attempts,^39^ and suicide deaths between 1993-2012,^40^ 2001-2015,^23^ and 1999-2015.^15^ Our results expand on previous reports of a disproportionate increase in suicide attempts among Black adolescents relative to other racial/ethnic groups with more updated data, investigation of turning points, and comparisons with trends in suicidal ideation.

Although few studies have examined the mechanisms underlying suicide attempts in Black adolescents,^41^ existing findings suggest structural inequalities and social determinants of health factors as plausible explanations.^41,42,43^ Black adolescents were more likely to experience long-standing socioeconomic inequalities (eg, poverty, racial segregation), living in families with financial hardships, and reporting other behavioral challenges.^15,44,45,46,47^ Such experiences could disproportionately precipitate greater disparities in untreated mental illness, levels of hopelessness, and risks of suicidal behaviors among Black youths.^35^ Notably, Black adolescents had higher adverse childhood experience (eg, abuse, neglect) exposures than their White and Hispanic counterparts,^48^ and adverse childhood experiences had a strong and dose-responsive association with attempted suicide among adolescents.^49^ Black adolescents also experienced greater online race-related traumatic events that may increase their risks of attempting suicide.^50,51^ More research on the race-specific risk and protective factors for suicide attempts among Black youths is needed.

The higher prevalence of suicidal ideation and suicide attempts in female compared with male adolescents indicates the need for the sex gaps to be addressed. Specifically, even with the observed decrease, prevalence rates in suicidal ideation and suicide attempts among female adolescents remained high in 2019 (Figure 3). Narrowing sex gaps were consistently observed in emergency department visits for suicidal ideation or nonfatal suicide attempts,^4,5,13,33^ suicide death,^12,52^ and self-reported suicidal ideation,^21,22,53^ with female adolescents aged 10 years or older more represented than males. The female-specific upward trends mirrored the trajectories of depression, with increasing depressive symptoms observed only among females since 2005.^22,37,54^ The post-2009 increasing trend in suicidal ideation among White female youths paralleled an increasing trend in mental health service use for suicidal ideation or suicide attempts among White female youths aged 12 to 17 years from 2009 to 2018.^55^

Adolescents in recent decades are experiencing rapid change in technology with behavioral consequences (eg, disrupted sleep, increased obesity) and evolving cultural norms associated with mental health discourse (eg, more suicide-related lyrics in popular rap music).^21,22,53,56^ Increasing suicidal ideation among female youths after 2007 calls for the need for suicide interventions addressing female-specific risks, particularly given their greater vulnerability to school bullying, cyberbullying, and peer victimization than male adolescents.^11,57,58,59^ Our results lend support to previous studies^35,60^ suggesting the need to consider both individual stress-diathesis risks and societal-contextual factors at large.

Previous suicide prevention efforts may contribute to short-term decreases in suicide attempts but are inefficient to prevent the upward trends that started in 2007 (eFigure 1 in the Supplement),^61,62^ especially among male and Black adolescents.^14^ Our study suggests that more funding support and policy advocacy are warranted to develop more comprehensive and culturally appropriate suicide prevention programs targeting different risks of ideation and attempts across sex and racial/ethnic groups. Future suicide risk assessments should avoid generalized solutions.^63^ Growing risks, such as adverse childhood experiences, systematic racism, discrimination, neighborhood violence, and socioeconomic disparities, need to be included in youth suicide prevention programs in general and for males and Black youths in particular.^12,23,40,41^ Barriers associated with access and adherence to mental health treatment at structural (eg, transportation, costs, availability of school-based mental health services), social (eg, stigma), and interpersonal (eg, implicit and explicit bias among clinicians toward racial minorities) levels should be addressed through interventions targeting access to mental health care and social determinants of health.^42^ Prevention approaches, including health promotion campaigns to increase the awareness of suicidal ideation, the intention to seek help from mental health professionals, coping skills, and the policies that strengthen financial stability, may improve suicide literacy and prevent youth suicide.^64^ Relative contributions of suicide prevention strategies (eg, risk assessment, public awareness, postvention), policies (eg, addressing the opioid crisis, bullying), and macrolevel societal factors (eg, economic context, structural racism) deserve attention.^24,65^

### Strengths and Limitations

A strength of this study is our use of large, nationally representative, most recent YRBS data across 1991-2019, which offers a unique opportunity to examine distinct trends in suicidal ideation and suicide attempts across sex and race/ethnicity among US adolescents. This study has limitations. First, the cross-sectional nature of YRBS limits the determination of causality in the trends. Second, YRBS only surveyed students who attend schools; thus, the data are not representative of all adolescents in the age group. Third, the measurement of suicidal behaviors and demographic characteristics are self-reported. Despite the good test-retest reliability^66^ and wide use in previous studies,^60^ there is a potential for self-reporting bias (eg, reluctance to disclose ideation and attempt history). The questions cannot capture short-term changes in suicidal thoughts and behaviors (eg, less than weekly). Future research that uses validated assessment instruments (eg, Columbia–Suicide Severity Rating Scale)^67^ or ecological momentary assessment technology that delivers questions on smartphones will increase understanding of more-imminent risks associated with suicide attempts among adolescents. Suicide deaths and underreporting of nonfatal suicide attempts may influence the results. Fourth, given the possible age-related racial/ethnic disparities observed in previous literature,^23^ further investigation on whether the trends in suicidal ideation and suicide attempts varied by grade levels is warranted to guide more targeted interventions for younger and older adolescents.

## Conclusions

The findings of this study suggest that addressing suicide disparities is a priority in research, clinical practice, and policy. We found large increases in the prevalence of suicide attempts in male and Black adolescents from 1991 through 2019. Female adolescents, particularly among White, Black, and Hispanic groups, experienced increasing suicidal ideation since 2009, but not nonfatal suicide attempts. From a policy perspective, our results highlight the need to implement evidence-based, structural, and suicide prevention programs involving collaboration between health systems and communities. Males, particularly Black male adolescents, appear to have the greatest need in terms of prevention of suicidal behaviors. Both groups have underperformed in help-seeking capacity and there may be stigma and racial barriers to getting psychiatric treatment for young Black males. Strategies that prioritize monitoring the trends in risk factors for suicidal behaviors in racial/ethnic subgroups, design culturally appropriate prevention programs, alleviate structural inequality, reduce mental health stigma and barriers to health care, and promote help-seeking should be ethnically and sexually diversified for effective suicide prevention.

## Notes

Supplement. **eFigure 1.** Turning Points in Temporal Trends in Suicidal Subgroups and Possible Associated Policy and Societal Factors, 1991-2019 **eFigure 2.** Prevalence of Suicidal Ideation and Suicide Attempts among US Adolescents, by Sex, 1991-2019 **eFigure 3.** Prevalence of Suicidal Ideation and Suicide Attempts among US Adolescents, by Race/ethnicity, 1991-2019 **eFigure 4.** Prevalence of Suicidal Ideation and Suicide Attempts among US Adolescents, by Sex and Race/ethnicity, 1991-2019 **eFigure 5.** Trends in Suicidal Ideation and Suicide Attempts among US Adolescents, by Sex and Race/ethnicity, 1991-2019
