# Supplementary material for: Temporal Trends in Suicidal Ideation and Attempts Among US Adolescents by Sex and Race/Ethnicity, 1991-2019
Source: JAMA Netw Open. 2021 Jun 14;4(6):e2113513. doi: 10.1001/jamanetworkopen.2021.13513 (PMC8204211; doi:10.1001/jamanetworkopen.2021.13513)
Supplement: Supplement. — eFigure 1. Turning Points in Temporal Trends in Suicidal Subgroups and Possible Associated Policy and Societal Factors, 1991-2019 eFigure 2. Prevalence of Suicidal Ideation and Suicide Attempts among US Adolescents, by Sex, 1991-2019 eFigure 3. Prevalence of Suicidal Ideation and Suicide Attempts among US Adolescents, by Race/ethnicity, 1991-2019 eFigure 4. Prevalence of Suicidal Ideation and Suicide Attempts among US Adolescents, by Sex and Race/ethnicity, 1991-2019 eFigure 5. Trends in Suicidal Ideation and Suicide Attempts among US Adolescents, by Sex and Race/ethnicity, 1991-2019 [file jamanetwopen-e2113513-s001.pdf]

Supplementary Online Content

Xiao Y, Cerel J, Mann JJ. Temporal trends in suicidal ideation and attempts among US adolescents by sex and race/ethnicity, 1991-2019. *JAMA Netw Open*. 2021;4(6):e2113513. doi:10.1001/jamanetworkopen.2021.13513

eFigure 1. Turning Points in Temporal Trends in Suicidal Subgroups and Possible Associated Policy and Societal Factors, 1991-2019
eFigure 2. Prevalence of Suicidal Ideation and Suicide Attempts among US Adolescents, by Sex, 1991-2019
eFigure 3. Prevalence of Suicidal Ideation and Suicide Attempts among US Adolescents, by Race/ethnicity, 1991-2019
eFigure 4. Prevalence of Suicidal Ideation and Suicide Attempts among U.S. Adolescents, by Sex and Race/ethnicity, 1991-2019
eFigure 5. Trends in Suicidal Ideation and Suicide Attempts among U.S. Adolescents, by Sex and Race/ethnicity, 1991-2019

This supplementary material has been provided by the authors to give readers additional information about their work.

© 2021 Xiao Y et al. *JAMA Network Open.*

eFigure 1. Turning Points in Temporal Trends in Suicidal Subgroups and Possible Associated Policy and Societal Factors, 1991-2019

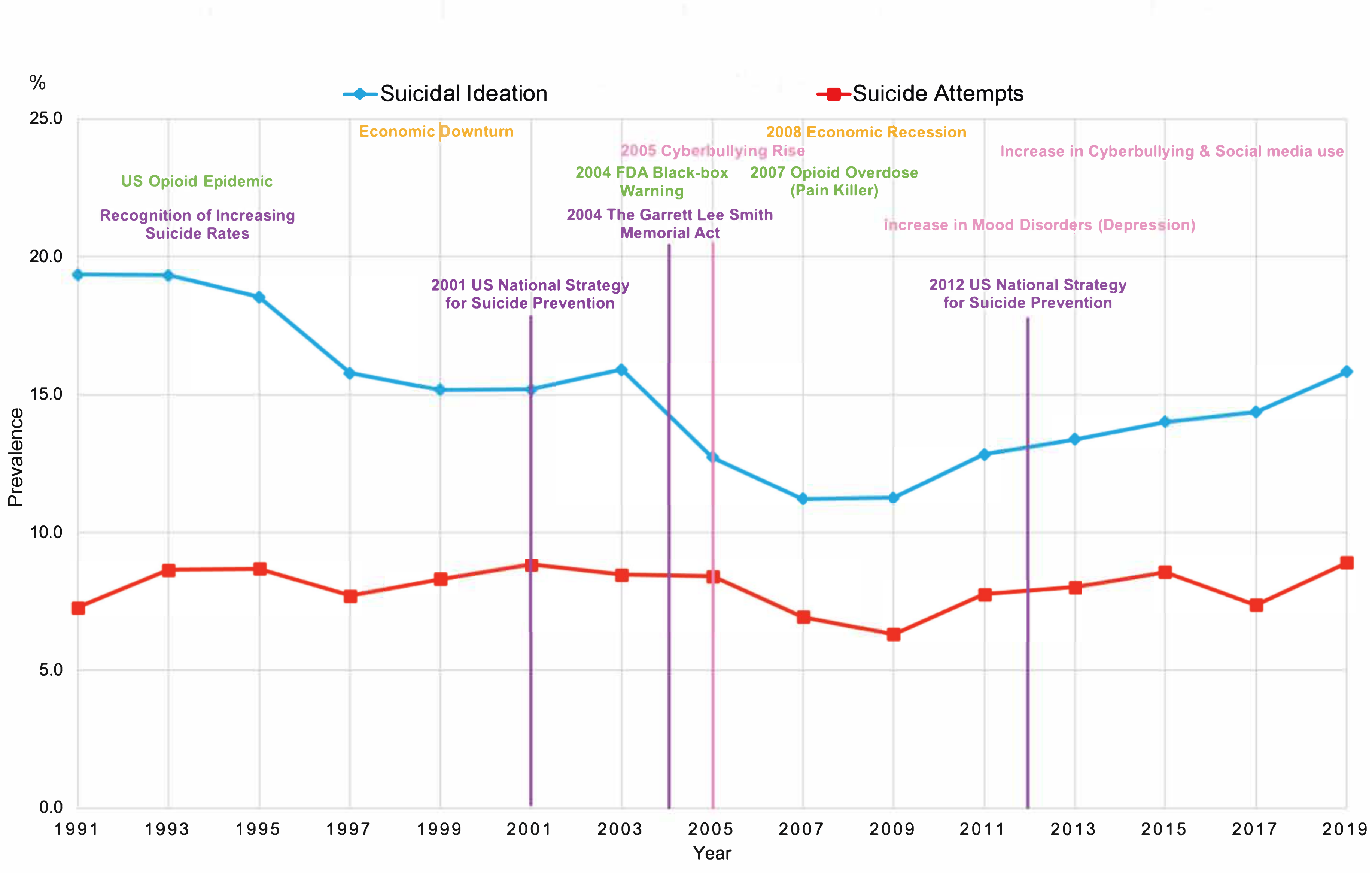

© 2021 Xiao Y et al. *JAMA Network Open.*

eFigure 2. Prevalence of Suicidal Ideation and Suicide Attempts among U.S. Adolescents, by Sex, 1991-2019

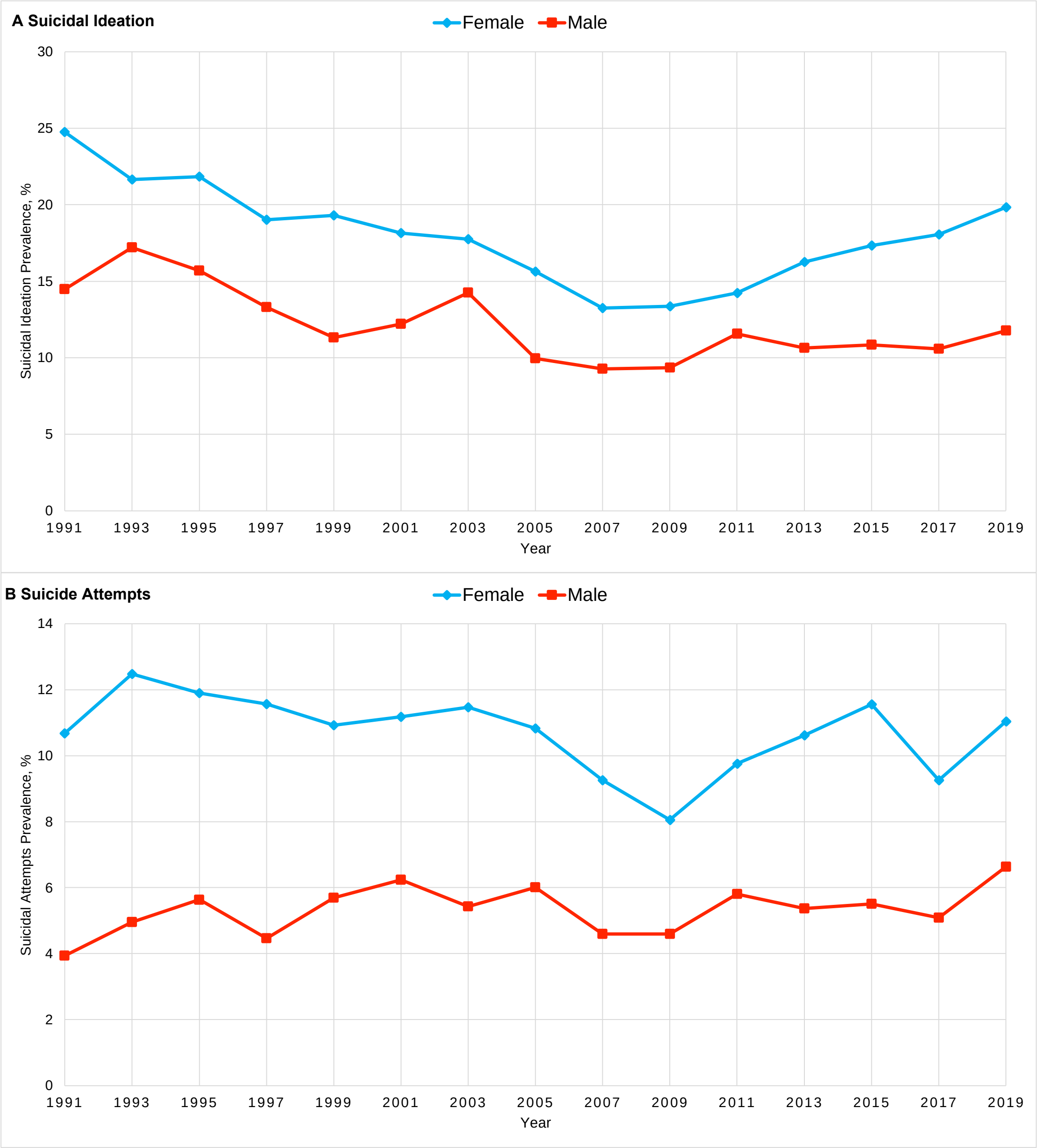

Note. For suicidal ideation, parallel comparisons by sex were not rejected (*P*=.55). For suicide attempts, APCs differed significantly from each other among females and males (*P*=.005).

© 2021 Xiao Y et al. *JAMA Network Open.*

eFigure 3. Prevalence of Suicidal Ideation and Suicide Attempts among U.S. Adolescents, by Race/ethnicity, 1991-2019

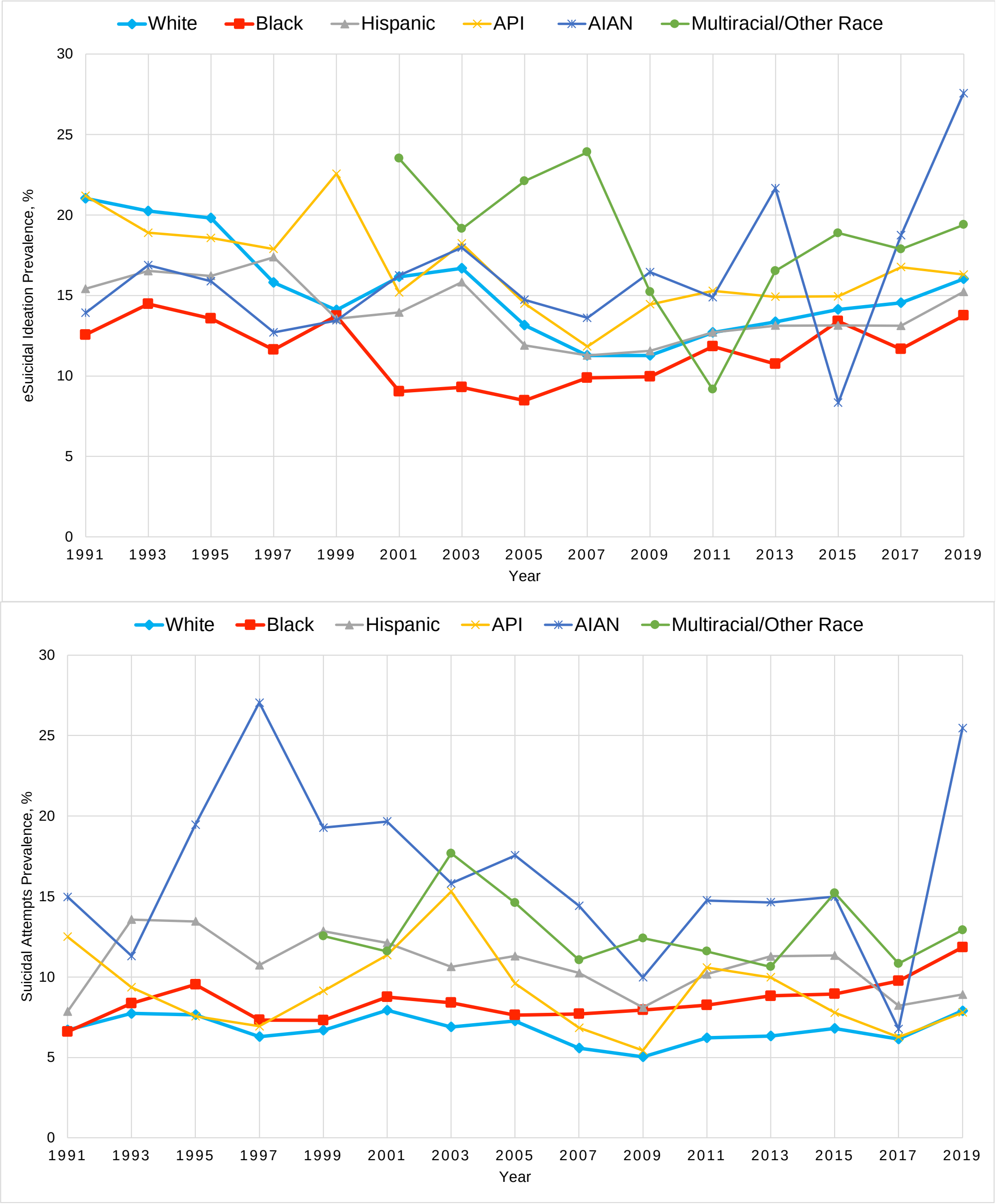

Note. For suicidal ideation, parallel comparisons were rejected when comparing White and Black adolescents (*P*=.01), White and AI/AN adolescents (*P*=.02),

© 2021 Xiao Y et al. *JAMA Network Open.*

Black and API adolescents (P=.02), and Hispanic and AI/AN adolescents (P=.02). For suicide attempts, APCs differed significantly between White and Black adolescents (P=.003) and Black and Hispanic adolescents (P=.01).

© 2021 Xiao Y et al. *JAMA Network Open.*

eFigure 4. Prevalence of Suicidal Ideation and Suicide Attempts among U.S. Adolescents, by Sex and Race/ethnicity, 1991-2019

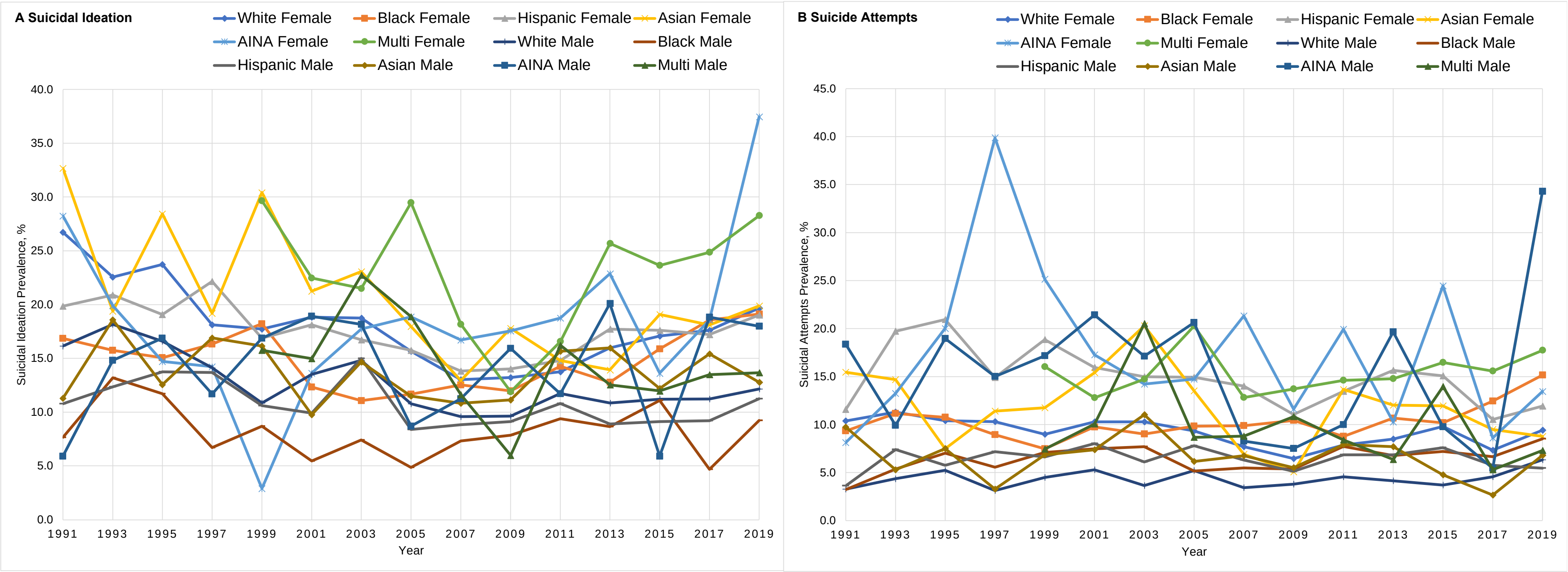

© 2021 Xiao Y et al. *JAMA Network Open.*

eFigure 5. Trends in Suicidal Ideation and Suicide Attempts among U.S. Adolescents, by Sex and Race/ethnicity, 1991-2019

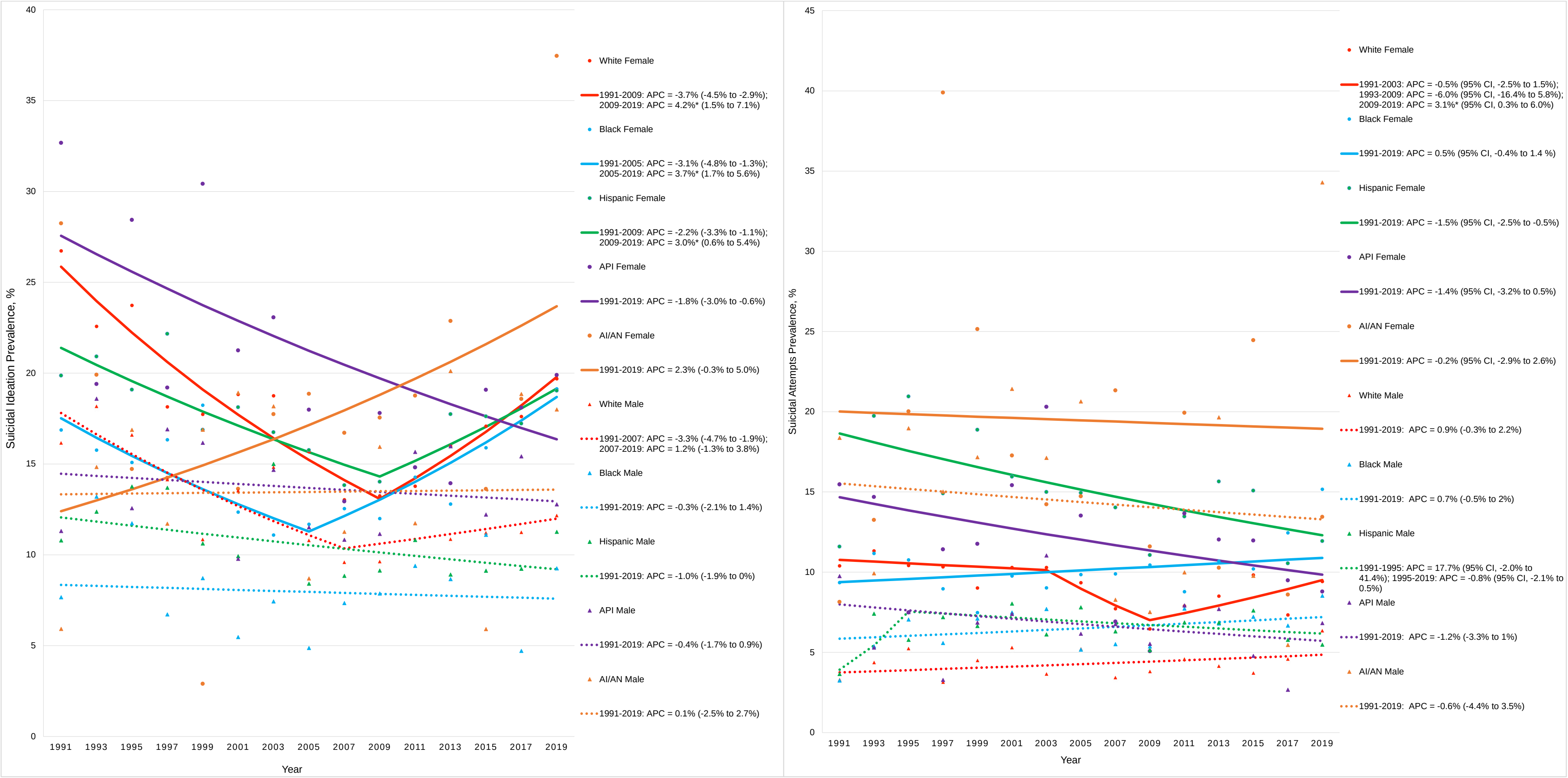

Note. For suicidal ideation, parallel comparisons were rejected when comparing White females vs. Black females (*P*=.02), White females vs. API females (*P*=.04), White females vs. AI/AN females (*P*=.03), Black vs. API females (*P*=.003), Black females vs. White males (*P*=.02), Black females vs. Hispanic males (*P*=.01), Hispanic female vs. AI/AN females (*P*=.01), Hispanic female vs. White males (*P*=.01), API female vs. AI/AN females (*P*=.02), AI/AN females vs. White males (*P*=.01), AI/AN female vs. Hispanic males (*P*=.01), White males vs. Black males (*P*=.04), and White males vs. API males (*P*=.03), indicating different pairwise slopes. For suicide attempts, parallel comparisons were rejected when comparing White females vs. Black females (*P*=.02), White females vs. White males (*P*=.02), White females vs. Black males (*P*=.01), White females vs. Hispanic males (*P*<.001), Black females vs. Hispanic females (*P*=.001), Black females vs. API females (*P*=.04), Black females vs. Hispanic males (*P*=.01), Hispanic female vs. White males (*P*=.01), Hispanic female vs. Black males (*P*=.01), Hispanic female vs. Hispanic males (*P*=.02), and API female vs. Hispanic males (*P*=.04), indicating the pairwise slopes were different. Results for non-Hispanic multiracial adolescents were not displayed due to missing values over time.

© 2021 Xiao Y et al. *JAMA Network Open.*
